# YvqE and CovRS of Group A *Streptococcus* Play a Pivotal Role in Viability and Phenotypic Adaptations to Multiple Environmental Stresses

**DOI:** 10.1371/journal.pone.0170612

**Published:** 2017-01-25

**Authors:** Amonrattana Roobthaisong, Chihiro Aikawa, Takashi Nozawa, Fumito Maruyama, Ichiro Nakagawa

**Affiliations:** 1 Section of Molecular Craniofacial Embryology, Graduate School of Medical and Dental Sciences, Tokyo Medical and Dental University, Tokyo, Japan; 2 Department of Microbiology, Graduate School of Medicine, Kyoto University, Kyoto, Japan; Ross University School of Medicine, DOMINICA

## Abstract

*Streptococcus pyogenes* (group A *Streptococcus*, or GAS) is a human pathogen that causes a wide range of diseases. For successful colonization within a variety of host niches, GAS utilizes TCSs to sense and respond to environmental changes and adapts its pathogenic traits accordingly; however, many GAS TCSs and their interactions remain uncharacterized. Here, we elucidated the roles of a poorly characterized TCS, YvqEC, and a well-studied TCS, CovRS, in 2 different GAS strain SSI-1 and JRS4, respectively. Deletion of *yvqE* and *yvqC* in JRS4 resulted in lower cell viability and abnormality of cell division when compared to the wild-type strain under standard culture conditions, demonstrating an important role for YvqEC. Furthermore, a double-deletion of *yvqEC* and *covRS* in SSI-1 and JRS4 resulted in a significantly impaired ability to survive under various stress conditions, as well as an increased sensitivity to cell wall-targeting antibiotics compared to that observed in either single mutant or wild-type strains suggesting synergistic interactions. Our findings provide new insights into the impact of poorly characterized TCS (YvqEC) and potential synergistic interactions between YvqEC and CovRS and reveal their potential role as novel therapeutic targets against GAS infection.

## Introduction

*Streptococcus pyogenes* (group A *Streptococcus*; GAS) is an important human pathogen that causes a wide spectrum of diseases, ranging from mild infections of the upper respiratory tract (pharyngitis) and skin (impetigo) to more serious invasive infections such as necrotizing fasciitis and streptococcal toxic shock syndrome (STSS) [1]. To successfully colonize and persist in a number of physiologically distinct host sites, GAS has developed complex mechanisms to cope with various environmental stresses. In many other gram-positive bacteria, sigma factors have been shown to play an essential role in the regulation of virulence genes in response to stress or growth [2]. However, GAS does not encode alternative sigma factors for regulating virulence gene expression [3]. Two-component systems (TCSs) are typically composed of two proteins, a sensor histidine kinase (HK) and its cognate response regulator (RR). TCSs are typically involved in various physiological functions in bacteria, such as survival, motility, metabolism, antibiotic resistance, stress response, and virulence, by sensing changes in the external or internal environment, modulating gene expression in response to a variety of stimuli inside the host cells, and avoiding host immune systems [4].

The available genome sequences indicate the presence of 12 cognate HK and RR pairs that are well conserved in all the strains of GAS [5]. Of these, the CovRS TCS is one of the most thoroughly studied regulatory systems due to its central role in the pathogenesis of GAS. In contrast to most TCSs, the results of several studies have indicated that the CovR/S TCS shows contradictory functions, such a Janus-like behaviour in the regulation of GAS virulence gene expression [6]. Under normal conditions, CovS acts in as a kinase to activate CovR and repress the expression of virulence factors but simultaneously acts as a phosphatase to permit gene expression in response to environmental changes during human infection. Moreover, CovR functions independently, and can still exert some of its regulatory function even in the absence of CovS [7]. CovR/S regulates the expression of about 15% of genes in the serotype M1 GAS genome [8] and mediates a general stress response to changing temperature, pH, and osmolarity [7]. In contrast to CovRS, most of TCSs in GAS, including YvqEC, remain functionally poorly characterized. To our knowledge, only a recent study has primarily focused on YvqE (HK) and reported that *yvqE* knockout (spy1622) in the GAS M1 clinical strain 1529, isolated from an STSS patient, resulted in growth reduction under acid conditions (pH 6.0) and reduced virulence in a mouse infection model [9]. However, relatively few have studied the YvqEC system in GAS, its physiological functions, underlying mechanisms, and cellular responses.

The YvqEC of GAS shows high sequence homology with LiaSR in *Bacillus subtilis* [10], *Streptococcus pneumoniae* [11], *Streptococcus mutans* [12, 13], *Streptococcus agalactiae* [14], and *Listeria monocytogenes* [15], with VraSR in *Staphylococcus aureus* [16, 17], and with CesSR in *Lactococcus lactis* [18]. Several studies have reported that the function of these systems seems to be a part of a complex regulatory network that counteracts cell envelope stress by maintaining its integrity under different stress conditions [10, 19], and that all of them are involved in species-specific responses to a variety of cell envelope-damaging agents, including cell wall-targeting antibiotics and antimicrobial peptides [20, 21]. Another recent study reported that deletion of either *yvqE* or *yvqEC* in *Bacillus thuringiensis* increases its susceptibility to vancomycin and suggested that YvqEC system plays a role in vancomycin resistance [22].

Strains SSI-1 and JRS4 are representative of GAS invasive and non-invasive strains, respectively. The invasive STSS strain SSI-1 (serotype M3) has been shown to contain a large-scale genomic rearrangement and differ from other GAS strains [23], suggesting that SSI-1 acquired strain-specific virulence factors. Moreover, the non-invasive isolate JRS4 (serotype M6) [24], is most widely used in studies of bacteria-host cell interactions [25, 26]. In the present study, we sought to characterize the phenotypes associated with a series of *yvqE*/*C* and *covR*/*S* TCSs mutants in the GAS strains SSI-1 and JRS4 in regards to the fundamental roles in each HK and RR gene, as well as their synergistic actions in double mutant strains. This study will provide a better understanding on the individual physiological roles of each gene, which should provide important new insights into the biological function of the YvqEC and CovRS systems.

## Materials and Methods

### Bacteria strains, culture media, and culture conditions

GAS strains used in this study are listed (S1 Table). Wild-type SSI-1 (serotype M3) was isolated from a patient with STSS in Japan in 1994 [23]. Wild-type JRS4 (serotype M6) is a streptomycin-resistant derivative of strain D471 [24]. Wild-type and mutant strains were grown at 37°C without agitation in Todd-Hewitt broth (BD Diagnostic Systems, Franklin Lakes, NJ) supplemented with 0.2% yeast extract (Nacalai Tesque, Kyoto, Japan) (THY). *Escherichia coli* strain DH10B was used as a host for plasmid construction. Strain DH10B was cultured in Luria-Bertani (LB) broth or LB agar (Nacalai Tesque, Kyoto, Japan) at 37°C. When necessary, antibiotics were used at the following concentrations: spectinomycin (Nacalai Tesque, Kyoto, Japan) at 100 μg/ml for *E*. *coli* and GAS; erythromycin (Nacalai Tesque, Kyoto, Japan) at 200 μg/ml and 0.5 μg/ml for *E*. *coli* and for GAS, respectively.

### Construction of deletion mutants

Deletion mutants were constructed by double-crossover recombination using the thermo-sensitive suicide vector pSET4S [27] carrying a spectinomycin-resistance gene. The 800-bp region upstream and 800-bp region downstream of each targeted gene were separately amplified by PCR from wild-type genomic DNA using the primer pairs target gene upper800fwd/target gene upper800rev and target gene down800fwd/target gene down800rev, respectively (S2 Table), and Gibson Assembly Mastermix (New England BioLabs, Ipswich, MA) was used to join the 2 DNA fragments with *SmaI*-digested pSET4s. Recombinant plasmids were transformed into *E*. *coli* DH10B competent cells, and plasmids of the positive colonies were purified using the High Pure Plasmid Isolation Kit (Roche, Basel, Switzerland). The plasmids were introduced into wild-type SSI-1 and JRS4 by electroporation, and mutants were selected on spectinomycin-containing plates at 28°C. Single-crossover chromosomal insertions were selected by shifting to the non-permissive temperature of 37°C, while maintaining spectinomycin selection. Single-crossover mutant colonies were passaged several times at 28°C without antibiotic selection, and spectinomycin-sensitive colonies were then screened for either gene deletion or return to the wild-type genotype by PCR using the target gene upper800fwd/ target gene down800rev primer pair. For constructing the Δ*yvqEC*Δ*covRS* double mutant, the Δ*covRS* mutant strain was used for the deletion of *yvqEC* following the same procedure as described above.

### Construction of complemented strains

Knock-in complemented strains were generated by reintroducing *yvqE*, *yvqC* or *yvqEC* and *covRS* into its original location (chromosomal restoration), the complete coding sequences including their 800-bp upstream and 800-bp downstream sequences, were amplified by PCR from wild-type genomic DNA using the target gene upper800fwd/target gene down800rev primer pair. PCR products were subsequently cloned into pSET4s, and the plasmids were transformed into the mutant strains by electroporation. Knock-in complementation followed the same deletion mutant procedure as described above. Successful restoration of the ORF in the knock-in strain was confirmed by PCR. To construct the complemented JRS4Δ*yvqE* and JRS4Δ*yvqC* strains by plasmids that overexpressed *yvqE* or *yvqC*, the complete coding sequences of *yvqE* and *yvqC* were amplified by PCR from JRS4 genomic DNA using the yvqE-1352_fwd/yvqE-1352_rev and yvqC-1351_fwd/yvqC-1351_rev primer pairs, respectively. PCR products were cloned into the pAT18 shuttle vector [28]. The plasmids were transformed by electroporation into JRS4Δ*yvqE* and JRS4Δ*yvqC* cells, and the complemented strains were selected on erythromycin-containing plates.

### Bacterial growth and viable cell count analyses

Wild-type and mutant strains were grown overnight in THY broth at 37°C. Then, 100 μl of pre-culture was added to 5 ml fresh THY. After 15 s of mixing, the turbidity of the culture was determined by measuring the optical density at 600 nm (OD_600_) every 30 min for 10 h using the WPA Colorwave colorimeter (model C07500; Biochrom, Cambridge, UK). Growth curves were constructed by plotting the OD_600_ values against time. Data were expressed as the mean and standard deviation from 3 independent experiments. To calculate the number of colony forming units (cfu/ml) at a given time, 100 μl culture aliquots were serially diluted and plated onto THY agar. Data were expressed as the mean and standard deviation from 3 independent experiments.

### Live/dead bacterial viability staining

Cell viability was estimated using LIVE/DEAD BacLight Bacterial Viability Kit (Molecular Probes, Eugene, OR) for microscopy observation. This kit differentially stains live or undamaged cells versus dead or damaged cells using 2 nucleic acid stains. SYTO 9 can label all bacterial cells whether live or dead, while propidium iodide (PI) enters only cells with damaged cell membranes. At an OD_600_ of 0.8, 2 ml of each bacterial culture was harvested by centrifugation and re-suspended with 1 ml phosphate-buffered saline (PBS). SYTO 9 (component A) and PI (component B) were mixed at a 1:1 ratio and 3 μl aliquots were added to each cell sample. The suspensions were incubated for 15 min at room temperature in the dark. Bacteria were visualized with Olympus Fluoview FV1000 confocal scanning laser microscope (Olympus, Tokyo, Japan). The experiments were repeated at least 3 times independently. Digital image analysis was performed using ImageJ processing software (US NIH). More than 80,000 stained-cells were counted in at least ten randomly chosen fields. The ratio of red fluorescence to green fluorescence was used to calculate cell death rates using the following equation: (the number of PI-stained cells/ the number of SYTO9-stained cells) × 100. Data were expressed as the mean and standard deviation.

### Vancomycin, PI, and 4’, 6-diamidino-2-phenylindone (DAPI) fluorescence staining

Septal cell wall synthesis was observed by staining with boron-dipyrromethene fluorescein-conjugated vancomycin (FL-van; Molecular Probes, Eugene, OR). At an OD_600_ of 0.8, 500 μl of each bacterial culture was stained directly by adding FL-van at a final concentration of 2 μg/ml and PI at a final concentration of 20 μg/ml for 15 min at room temperature, followed by DAPI (Dojin Chem, Tokyo, Japan) to label chromosomal DNA at a final concentration of 0.2 μg/ml for 10 min at room temperature. Images are visualized with Olympus Fluoview FV1000 confocal scanning laser microscope. The experiments were independently repeated at least 3 times.

### Electron microscopy

Bacterial pellets grown in THY broth at 37°C to an OD_600_ of 0.8 were fixed in 1% osmiumtetroxide in 30 mM HEPES at 4°C for 2 h. Pellets were then post-fixed with 2.5% glutaraldehyde and 4% tannic acid in 30 mM HEPES at 4°C overnight, and embedded in agar-gel. After washed with HEPES, samples were fixed in 1% osmiumtetroxide in 30 mM HEPES at 4°C overnight. The samples were then stained with 0.5% uranyl acetate at room temperature for 1 h, dehydrated through graded ethanol series, embedded in epoxy-resin Luveak (Nacalai Tesque, Kyoto, Japan) and polymerized at 60°C for 3 days. The samples were cut into ultrathinsections (70 nm) on an ultramicrotome (EM UC6; Leica). The ultrathinsections were mounted on mesh grids and stained with uranyl acetate and lead citrate and examined with electron microscopic H-7650 (Hitachi, Tokyo, Japan).

### Quantitative reverse transcription-PCR (qRT-PCR) and RNA extraction

Three independent cultures of each selected strain were grown to OD_600_ of 0.8 at 37°C in THY broth. For RNA extraction, bacteria were first centrifuged and pellets were treated with Max Bacterial Enhancement Reagent (Invitrogen, Paisley, UK), and then lysed using TRIzol LS (Invitrogen, Paisley, UK). Afterwards, RNA was treated with TURBO DNase (Life Technologies, Carlsbad, CA). The RNA concentration and purity was determined using a nanodrop. qRT–PCR was performed in triplicate using separate RNA extractions (100 ng/reaction) with Quantifast SYBR Green RT-PCR kit (Qiagen, Hilden, Germany) on a LightCycler Nano (Roche, Burgess Hill, UK) according to the manufacturer’s instruction. Normalized expression levels of the target gene transcripts were calculated relative to the housekeeping gene, *proS* [29] using the 2^−ΔΔ^*CT* method [30].

### Stress tolerance assays

To evaluate the ability of GAS strains to grow under various stress conditions, 100 μl of each bacterial culture was added to 5 ml fresh THY and subjected to different stress conditions such as osmotic stress (addition of NaCl to a final concentration of 0.5 M), oxidative stress (addition of H_2_O_2_ to a final concentration of 1 mM), acidic stress (pH adjustment to 5.5 with HCl), and heat stress (incubation at 42°C). After 15 s of mixing, the turbidity of culture was determined by measuring the OD_600_ values every 1 h using the WPA Colorwave colorimeter. Data were expressed as the mean and standard deviation from 3 independent experiments. To calculate the number of CFUs (cfu/ml) at a given time after stress exposure, 100 μl culture aliquots were serially diluted and plated onto THY agar. The results were normalized to the CFUs at time zero using the following equation: (CFUs at indicated time/CFUs at time zero) × 100. Data were expressed as the mean and standard deviation from 3 independent experiments in triplicate.

### Bacterial cell lysis assays

Triton X-100, a non-ionic detergent that induces cell lysis, was used for this assay. GAS cells were grown in THY broth at 37°C to an OD_600_ of 0.8 and collected by centrifugation. The pellets were re-suspended with 50 mM Tris-HCl (pH 7.4) containing 0.1% Triton X-100 (Nacalai Tesque, Kyoto, Japan). A 200-μl aliquot of each cell suspension was added into a 96-well plate and incubated at 37°C. Cellular lysis was monitored by measuring the decrease in OD_600_ of the cell suspensions every 1 h for 4 h with an iMark microplate absorbance reader (Bio-Rad, Hercules, CA). The percent initial turbidity was calculated by following equation: (OD_600_ at indicated time/OD_600_ at time zero) × 100. Lower percent initial turbidity values reflect increase cell lysis. Data were expressed as the mean and standard deviation from 3 independent experiments in triplicate.

### Biofilm formation analysis by crystal violet staining

Overnight cultures of wild-type and mutant strains in THY broth were diluted ten-fold in C medium (0.5% proteose peptone, 1.5% yeast extract, 10 mM K_2_HPO_4_, 0.4 mM MgSO_4_, and 17 mM NaCl; pH 7.5), and 200-μl aliquots were inoculated into 96-well polystyrene flat bottom plates (TPP, Trasadingen, Switzerland) and incubated at 37°C for 24 h. After removing the medium, biofilms were gently washed 3 times with PBS. The wells were stained with 100 μl of 0.2% (w/v) crystal violet (Nacalai Tesque, Kyoto, Japan) solution at room temperature for 10 min and gently washed 3 times with PBS. The bound dye was extracted from the stained cells with 100 μl of 1% sodium dodecyl sulphate. Biofilms were then quantified by measuring the OD_560_ values using iMark microplate absorbance reader. Wells incubated without bacteria were used as blanks. The OD_560_ value from the blank well was subtracted from the test values. Data were expressed as the mean and standard deviation from 3 independent experiments in quadruplicate wells.

### Antimicrobial susceptibility assay

The minimal inhibitory concentration (MIC) of penicillin G (Nacalai Tesque, Kyoto, Japan), bacitracin (Sigma-Aldrich, St. Louis, MO), and nisin (MoBiTec, Göttingen, Germany) was determined using the broth microdilution method. Bacterial pre-cultures were inoculated into THY broth and grown at 37°C to an OD_600_ of 0.8. Then, 10-μl aliquots were inoculated into 96-well plates, containing 200 μl of THY broth supplemented with 2-fold serial dilutions of antibiotics in each well. The cultures were incubated at 37°C for 18 h, and the bacterial turbidity was measured using an iMark microplate absorbance reader. MIC was defined as the lowest concentration of antibiotic that completely inhibited cell growth. The experiments were independently repeated 3 times.

### Measurement of cell-associated hyaluronic acid (HA)

Bacterial pre-cultures were inoculated into THY broth and grown at 37°C to an OD_600_ of 0.8. A 100-μl aliquot from each bacterial culture was used for determining the number of CFUs (cfu/ml) by colony counting on a THY agar plate. The cells were then centrifuged and re-suspended in 500 μl sterile distilled water. The HA capsule was released into the aqueous phase by adding 1 ml chloroform and shaking the mixture vigorously for 1 h at room temperature. The aqueous phase was then separated by centrifugation. HA content was determined in the aqueous phase by measuring the OD_640_ values after adding an equal volume of 0.02% (w/v) Stains-All (Sigma-Aldrich, St. Louis, MO), 0.06% glacial acetic acid, 50% formamide (Nacalai Tesque, Kyoto, Japan). Absorbance values were compared with a standard curve generated by the known concentration of HA (Nacalai Tesque, Kyoto, Japan). The amount of cell-associated HA was expressed as femtograms per cfu (fg/cfu) and standard deviation from 3 independent experiments.

### Bacterial internalization assay (gentamicin protection assay)

HeLa cells were maintained in Dulbecco’s modified Eagle’s medium (DMEM; Nacalai Tesque, Kyoto, Japan) supplemented with 10% fetal bovine serum (FBS; JRH Biosciences, Lenexa, KS) and 50 μg/ml gentamicin (Nacalai Tesque, Kyoto, Japan) in a 5% CO_2_ incubator at 37°C. To assay bacterial internalization, HeLa cells were seeded into 24-well tissue culture plates (4 × 10^4^ cells/well) and cultured at 37°C in 5% CO_2_ overnight. GAS was grown to an OD_600_ of 0.8 in THY and then used to infect HeLa cell cultures at a multiplicity of infection (MOI) of 100 (4 × 10^6^ cfu/well) without antibiotics for 1 h. Thereafter, wells were thoroughly washed by PBS to remove unbound bacteria before adding antibiotics (100 μg/ml gentamicin and 100 U/ml penicillin G), incubated for 1 h to kill extracellular bacteria. Then, cells were washed and lysed in 1 ml sterile distilled water, and serial dilutions were placed on THY agar plates to enumerate CFU. The number of internalized GAS was calculated as the percentage of bacterial cells remaining after gentamicin treatment relative to cell-associated bacteria before gentamicin treatment. The experiments were performed in triplicate with 3 independent experiments.

### Lactate dehydrogenase (LDH) cytotoxicity assay

Cytotoxicity on HeLa cells was evaluated by measuring the release of lactate dehydrogenase (LDH) using a LDH cytotoxicity detection kit (Takara Bio Inc., Shiga, Japan) according to manufacturer’s instructions. Briefly, HeLa cells were seeded into 96-well tissue culture plates at densities 2 × 10^4^ cells per well and cultured at 37°C in 5% CO_2_ for overnight. Bacteria at an OD_600_ of 0.8 were added to cell cultures at a multiplicity of infection (MOI) of 100 without antibiotics for 1 h. At 4 h after infection, the release of LDH into culture supernatant was measured in the iMark microplate absorbance reader (Bio-Rad). The percentage of cytotoxicity was calculated. Data were expressed as the mean and standard deviation from 3 independent experiments.

### Blood survival assay

To confer phagocytosis resistance, bacterial survival in whole blood was assessed using standard Lancefield whole-blood bactericidal assay [31]. Briefly, blood from 3 healthy human volunteers was collected using a heparin tube (BD Vacutainer; Becton Dickinson, East Rutherford, NJ) and immediately used in this assay. Bacterial pre-cultures were grown in THY broth at 37°C to an OD_600_ of 0.8, and a 50-μl aliquot of diluted bacteria in PBS, containing about 10^4^ cfu, was added to 450 μl heparinized blood. The tubes were rotated with end-over-end at 37°C for 3 h, and serial dilutions of the mixtures were plated on THY agar. Bacterial growth (multiplication factor) was calculated by dividing the number of CFUs after 3 h of incubation by CFUs in the original inoculum. Each strain was tested in 3 individually collected blood samples. Data were expressed as the mean and standard deviation from 3 independent experiments. This study was performed in accordance with the protocol and guidelines approved by the ethics committees of Kyoto University Graduate School of Medicine, Japan (Approved Number: R0100) and written informed consent was obtained from all volunteers.

### Statistical analysis of data

Data were expressed as the mean and standard deviation from at least 3 independent experiments. The differences between wild-type and the corresponding mutant strains were identified using unpaired 2-tailed *t* test.

## Results

### Construction and growth characterization of deletion mutants

**Fig 1:**
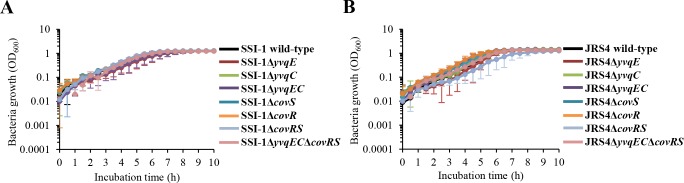
Growth curves of GAS under standard culture conditions (THY, 37°C). Growth curves of (A) SSI-1 wild-type and the corresponding mutant strains, and (B) JRS4 wild-type and the corresponding mutant strains. Data were expressed as the mean and standard deviation from 3 independent experiments.

To gain insight into the role of *yvqEC* and *covRS* TCSs in GAS, non-polar deletion mutants of Δ*yvqE*, Δ*yvqC*, Δ*yvqEC*, Δ*covS*, Δ*covR*, Δ*covRS*, and Δ*yvqEC*Δ*covRS* were independently constructed in wild-type SSI-1 and JRS4. We initially examined the growth characteristics of wild-type and mutant strains by measuring the turbidity of THY broth cultures. Although the difference was not statistically significant between the wild-type and the corresponding mutant strains, the SSI-1Δ*yvqE*, SSI-1Δ*yvqEC*, and JRS4Δ*covRS* strains exhibited slightly slower growth rate as compared with the wild-type strain, (Fig 1A and 1B). In addition, the standing overnight THY broth cultures of SSI-1Δ*yvqE* and JRS4Δ*yvqE* were visibly clear with bacterial cell sediment compared with wild-type strains, whereas other mutants were homogeneously turbid (S1 Fig).

### YvqE and YvqC are important for cell viability in JRS4 under standard culture conditions

We counted the number of cfu/ml on THY agar plates because increasing OD_600_ values (turbidity) in broth cultures reflects an increase in microbial cell mass, which includes both viable and dead cells. The number of cfu/ml of JRS4Δ*yvqE* and JRS4Δ*yvqC* was 1- to 2-log lower at an OD_600_ of 0.3 and of 0.8 than that of wild-type JRS4 (Table 1). These results indicated that JRS4Δ*yvqE* and JRS4Δ*yvqC* apparently grew as well as wild-type JRS4, but they had decreased viability. However, the JRS4Δ*yvqE* and JRS4Δ*yvqC*-complemented strains had similar viabilities to that of wild-type JRS4 (S2A and S2B Fig), indicating that the observed changes were caused by the deletion of *yvqE* or *yvqC*.

**Table 1:** Cell viability of wild-type and mutants produced in SSI-1 and JRS4 at OD_600_ of 0.3 and of 0.8 under standard culture conditions (THY, 37°C).

Strain	Colony count (cfu/ml)^a^	
	OD_600_ 0.3	OD_600_ 0.8
Wild-type SSI-1	2.4 x 10^8^ ± 5.5 x 10^7^	6.6 x 10^8^ ± 1.8 x 10^8^
SSI-1Δ*yvqE*	1.5 x 10^8^ ± 7.1 x 10^7^	5.3 x 10^8^ ± 1.2 x 10^8^
SSI-1Δ*yvqC*	3.0 x 10^8^ ± 1.0 x 10^8^	8.9 x 10^8^ ± 1.0 x 10^8^
SSI-1Δ*yvqEC*	1.8 x 10^8^ ± 8.5 x 10^7^	7.9 x 10^8^ ± 1.7 x 10^8^
SSI-1Δ*covS*	1.5 x 10^8^ ± 5.1 x 10^7^	7.2 x 10^8^ ± 2.8 x 10^8^
SSI-1Δ*covR*	1.7 x 10^8^ ± 5.6 x 10^7^	7.2 x 10^8^ ± 2.4 x 10^7^
SSI-1Δ*covRS*	1.8 x 10^8^ ± 8.7 x 10^7^	7.5 x 10^8^ ± 1.9 x 10^8^
SSI-1Δ*yvqEC*Δ*covRS*	**8.0 x 10**^**7**^ **± 1.4 x 10**^**7**^	5.0 x 10^8^ ± 7.8 x 10^7^
Wild-type JRS4	1.8 x 10^8^ ± 6.0 x 10^7^	4.8 x 10^8^ ± 1.3 x 10^8^
JRS4Δ*yvqE*	**1.4 x 10**^**6**^ **± 2.6 x 10**^**5**^	**8.1 x 10**^**6**^ **± 2.5 x 10**^**6**^
JRS4Δ*yvqC*	**4.2 x 10**^**7**^ **± 2.8 x 10**^**7**^	**2.5 x 10**^**8**^ **± 9.6 x 10**^**7**^
JRS4Δ*yvqEC*	**3.2 x 10**^**7**^ **± 3.0 x 10**^**7**^	3.7 x 10^8^ ± 2.5 x 10^8^
JRS4Δ*covS*	2.0 x 10^8^ ± 4.1 x 10^7^	6.9 x 10^8^ ± 2.4 x 10^8^
JRS4Δ*covR*	1.6 x 10^8^ ± 3.9 x 10^7^	7.2 x 10^8^ ± 2.6 x 10^8^
JRS4Δ*covRS*	2.4 x 10^8^ ± 1.5 x 10^8^	6.3 x 10^8^ ± 8.1 x 10^7^
JRS4Δ*yvqEC*Δ*covRS*	1.3 x 10^8^ ± 3.9 x 10^7^	6.2 x 10^8^ ± 1.3 x 10^8^

^*a*^ Data were expressed as the mean and standard deviation from at least 3 independent experiments. Bold indicates statistically significant differences between the wild-type and the mutant strain at *P* < 0.01 as determined by *t*-test.

**Fig 2:**
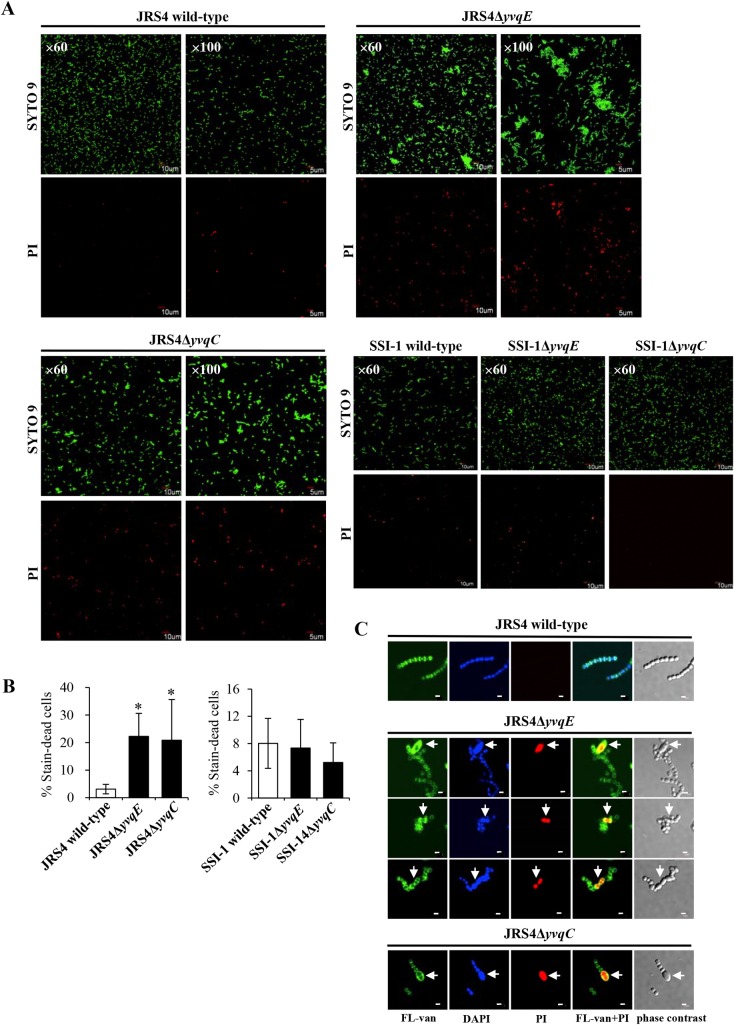
Deletion of *yvqE* and *yvqC* in JRS4 results in reduced viability. (A) Representative images of LIVE/DEAD-stained wild-type JRS4, JRS4Δ*yvqE*, JRS4Δ*yvqC*, wild-type SSI-1, SSI-1Δ*yvqE*, and SSI-1Δ*yvqC* grown in THY at an OD_600_ of 0.8. Live bacteria are stained green (SYTO 9) and dead bacteria are stained red (PI). Magnification, ×60 and ×100. (B) The percentage of dead cells in Δ*yvqE* and Δ*yvqC* strains was compared with wild-type. Data were expressed as the mean and standard deviation. Asterisks indicate statistically significant difference at *P* < 0.05 (*). (C) Representative images of wild-type JRS4, JRS4Δ*yvqE*, and JRS4Δ*yvqC* grown in THY at an OD_600_ of 0.8. Septum and cell wall of bacteria are stained green (FL-van), dead bacteria are stained red (PI), and nuclei of bacteria are stained blue (DAPI). Scale bars represent 1 μm. Arrows indicate defects in cell division.

JRS4Δ*yvqE* and JRS4Δ*yvqC* underwent a large decrease in the number of viable cells at an OD_600_ of 0.3 and of 0.8. To further confirm this observation, both JRS4Δ*yvqE* and JRS4Δ*yvqC* at an OD_600_ of 0.8 were stained with LIVE/DEAD reagents. Confocal microscopy images showed that more dead cells were found in JRS4Δ*yvqE* and JRS4Δ*yvqC* when compared to wild-type JRS4 (Fig 2A). A quantification analysis of the PI-stained cells confirmed a significant difference (*P <* 0.05) between mutant and wild-type JRS4 (Fig 2B). Moreover, it was clear that JRS4Δ*yvqE* and JRS4Δ*yvqC* cells exhibited increased bacterial clumps, forming aggregates (clusters of green fluorescein), unlike the wild-type strain, for which the cells were dispersed separately throughout the culture (Fig 2A), while complementation restored aggregation to levels similar to the wild-type (S2C Fig). JRS4Δ*yvqE* and JRS4Δ*yvqC* showed a marked increase in dead cells (10–20%), as determined using fluorescent dyes (Fig 2B), in spite of a viability loss of 1–2 log10 cfu/ml (Table 1). These numbers were not directly proportional because JRS4Δ*yvqE* and JRS4Δ*yvqC* also had a tendency to form aggregate cells and each aggregate produced a single colony. However, no differences were observed in the number of dead cells between SSI-1Δ*yvqE* and SSI-1Δ*yvqC*, and wild-type SSI-1. These resulted suggested that *yvqE* and *yvqC* function in a strain-specific manner.

**Fig 3:**
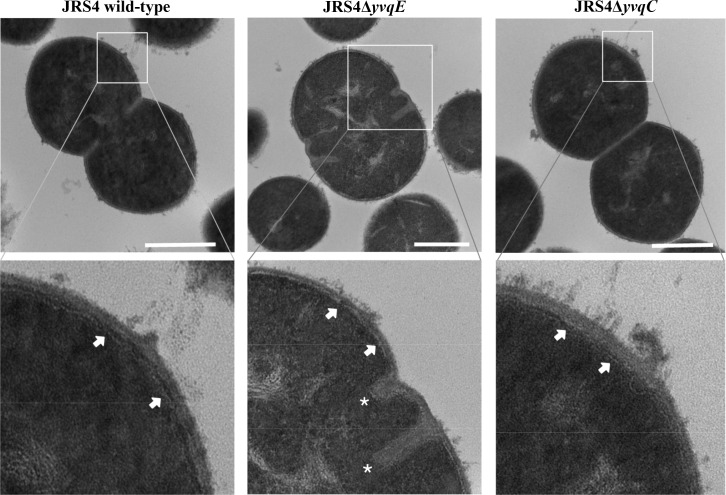
Transmission electron microscope (TEM) images of JRS4 wild-type, JRS4Δ*yvqE* and JRS4Δ*yvqC*. Cell wall architecture and septum formation were visualized by TEM on JRS4 wild-type, JRS4Δ*yvqE* and JRS4Δ*yvqC*. Arrows indicate peptidoglycan layer (clear black line adjacent to the edge of the cytoplasmic membrane), and asterisks indicate aberrant division septa. Scale bars represent 500 nm.

To reveal the growth abnormalities of JRS4Δ*yvqE* and JRS4Δ*yvqC*, we evaluated septal cell wall synthesis using FL-van. In wild-type JRS4, FL-van staining clearly depicted normal cell division and symmetrical septum formation, while DAPI staining showed a normal distribution of nucleoids (Fig 2C). In JRS4Δ*yvqE* and JRS4Δ*yvqC*, FL-van and DAPI staining suggested an alteration in cell division, since they exhibited aberrant cell morphology, and nucleoids in enlarged and bulged cells (Fig 2C). Furthermore, JRS4Δ*yvqE* and JRS4Δ*yvqC* division defective cells continued to maintain their shape, but were non-viable (Fig 2C). Additionally, transmission electron microscopy was used to analyse the morphology of the bacterial cell wall and septa. JRS4Δ*yvqE* and JRS4Δ*yvqC* cells had a thin peptidoglycan layer when compared with that of the JRS4 wild-type cells (Fig 3). In addition, some JRS4Δ*yvqE* cells exhibited abnormal division septa. These aberrant division events explained the cell aggregation phenotype. The effects of JRS4Δ*yvqE* and JRS4Δ*yvqC* on the transcription of cell wall synthesis- and cell division-related genes were assessed by qRT-PCR. The mRNA levels of *pbp1B* and *ftsL* genes were significantly downregulated (*P <* 0.05) by more than 2-fold in JRS4Δ*yvqE* and JRS4Δ*yvqC* cells (S3 Table). These data indicated that the YvqEC system was involved in GAS cell wall synthesis and division.

### The synergistic effects of YvqEC and CovRS systems are important for GAS adaptation and survival under stress conditions

**Fig 4:**
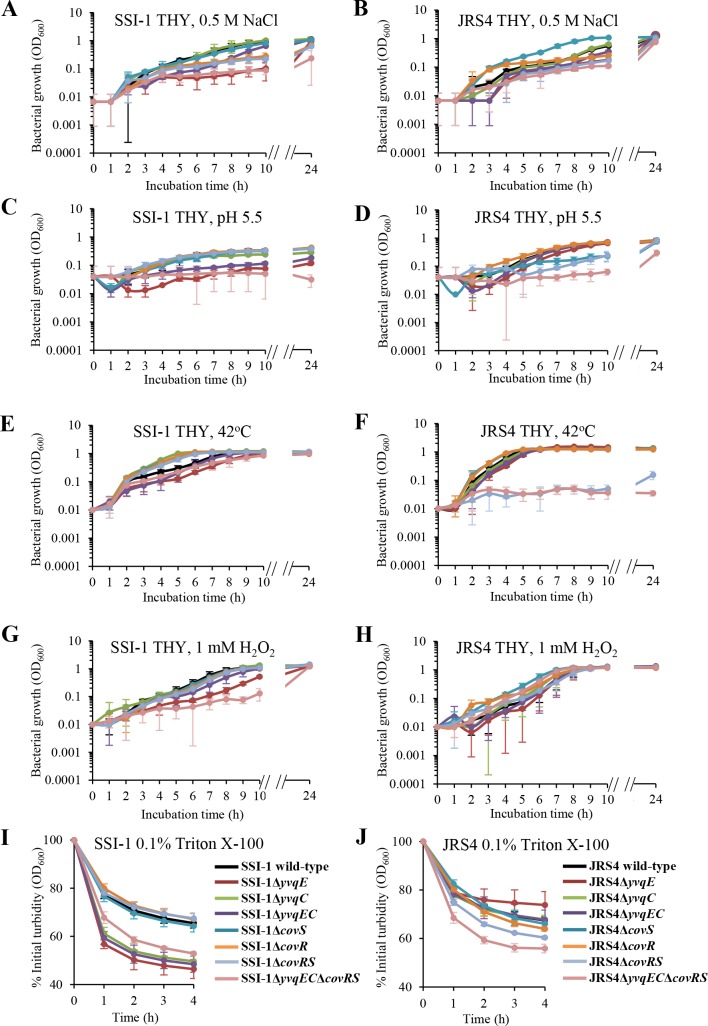
SSI-1 and JRS4 wild-type and the corresponding mutant strains grown under various stress conditions. SSI-1 and JRS4 wild-type and the corresponding mutant strains from overnight cultures were inoculated into fresh THY broth and grown under various stress conditions (A–H) for 24 h. The turbidity of SSI-1 and JRS4 wild-type and the corresponding mutant strains were measured at OD_600_ under (A, B) osmotic stress, (C, D) acidic stress, (E, F) heat stress, and (G, H) oxidative stress. Data were expressed as the mean and standard deviation from 3 independent experiments. Cellular lysis of (I) SSI-1 wild-type and the corresponding mutant strains and (J) JRS4 wild-type and the corresponding mutant strains in the presence of 0.1% Triton X-100 was monitored by recording the decrease at OD_600_. Data were normalized to the OD_600_ at time zero and was shown as the mean and standard deviation of triplicate wells from 3 independent experiments.

To investigate whether *yvqEC* and *covRS* in GAS are actively involved in the cellular stress response, wild-type and mutant strains were examined upon exposure to osmotic, oxidative, acidic, and heat stress. As expected, wild-type and mutant strains did not exhibit obvious differences of growth defects when grown under standard culture conditions (Figs 1A and 1B), while several mutants showed a growth delay when grown under stress conditions (Fig 4A–4H). The growth of SSI-1Δ*yvqE* was delayed for more than 10 h compared with that of wild-type SSI-1 under osmotic, acidic, and oxidative stress conditions (Fig 4A, 4C and 4G). The growth of JRS4Δ*covRS* was delayed for more than 10 h compared with that of wild-type JRS4 under osmotic, acidic, and heat stress conditions (Fig 4B, 4D and 4F). Importantly, the SSI-1 and JRS4 Δ*yvqEC*Δ*covRS* double mutant demonstrated a much slower growth than the wild-type or single mutant in almost all stress conditions. Complementation of *yvqEC* and *covRS* in the double mutant restored their growth ability to the wild-type levels (S3A–S3F Fig). In addition, SSI-1Δ*yvqE*, SSI-1Δ*yvqC*, SSI-1Δ*yvqEC*, SSI-1Δ*yvqEC*Δ*covRS*, JRS4Δ*covRS*, and JRS4Δ*yvqEC*Δ*covRS* were significantly more susceptible (*P* < 0.01) to Triton X-100 at all time points than wild-type SSI-1 and JRS4 (Fig 4I and 4J).

**Fig 5:**
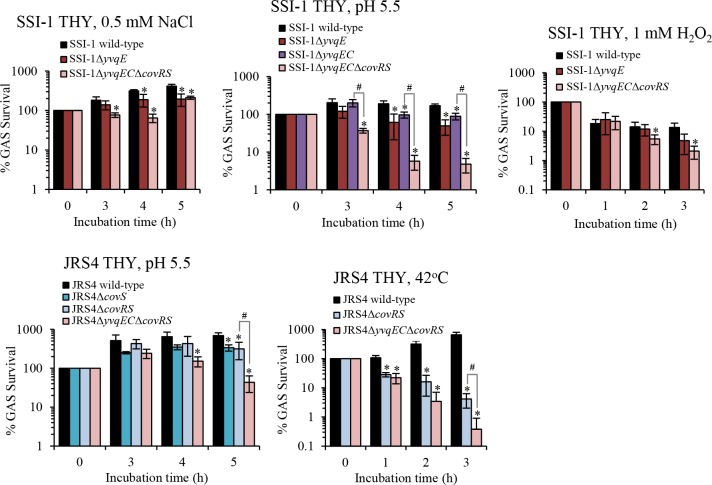
Survival of GAS after exposure to stress conditions. SSI-1 and JRS4 wild-type strains, and their corresponding mutant strains from overnight cultures were inoculated into fresh THY containing various stress-generating agents. An aliquot from each bacterial culture was used for the determination of the number of cfu/ml by colony counting on THY agar plates. The results were normalized to the OD_600_ at time zero, and percent survival at the indicated time was calculated. All results were shown as the mean with standard deviation of 3 independent experiments performed in triplicate. Asterisks indicate statistically significant differences compared to the results for the wild-type strain. *P < 0.05. Sharps indicate statistically significant differences between Δ*yvqEC* or Δ*covRS* strain, and Δ*yvqEC*Δ*covRS* strain: **#***P* < 0.05, as determined by *t*-test.

We further tested mutant strains with a severe growth defect by performing bacterial survival assays under the same stress conditions at indicated time points compared with wild-type strains. The survival rate of the tested mutant strains was significantly reduced (*P* < 0.05) compared with that of wild-type strains (Fig 5). Interestingly, the Δ*yvqEC*Δ*covRS* double mutant strain was significantly more sensitive than the single mutant Δ*yvqEC* or Δ*covRS* (*P* < 0.05), suggesting that the YvqEC and CovRS act synergistically and play an important role for GAS adaptation and survival under stress conditions.

### Synergistic effect of YvqEC and CovRS systems are involved in tolerance to cell wall targeting antibiotics

YvqEC and CovRS showed increased sensitivity to various stressors and Triton X-100-induced cell lysis. We therefore hypothesized that YvqEC and CovRS may affect the susceptibility to cell wall targeting antibiotics. To evaluate this hypothesis, the susceptibility of wild-type and mutant strains was verified with a panel of 3 cell wall targeting antibiotics; penicillin G, bacitracin, and nisin. MICs were determined as shown in Table 2. JRS4Δ*yvqE*, JRS4Δ*yvqC*, JRS4Δ*yvqEC*, and JRS4Δ*yvqEC*Δ*covRS* were found to be 2-fold more susceptible to penicillin G compared with wild-type JRS4. The MICs of bacitracin and nisin for SSI-1 and JRS4 Δ*yvqE*, Δ*yvqC*, Δ*yvqEC* were decreased 2-fold relative to the wild-type strains. The SSI-1Δ*yvqEC*Δ*covRS* double mutant was extremely sensitive to bacitracin (8-fold decrease in the MIC relative to the wild-type strain and 4-fold lower than that for the Δ*yvqEC* and Δ*covRS* single mutants). The JRS4Δ*yvqEC*Δ*covRS* exhibited 2-fold and 4-fold decrease in nisin MIC relative to those for JRS4Δ*yvqEC* and wild-type JRS4, respectively. Thus, there is a synergistic effect on antibiotic resistance, since the Δ*yvqEC*Δ*covRS* double mutant confer lower MICs than either Δ*yvqEC* or Δ*covRS*. Complementation of *yvqEC* and *covRS* in the double mutant restored the phenotype to the wild-type levels (data not shown).

**Table 2:** The minimal inhibitory concentration (MIC) of SSI-1 and JRS4 wild-type and the corresponding mutant strains.

Strain	MIC^a^ (μg/ml)		
	Penicillin G	Bacitracin	Nisin
Wild-type SSI-1	0.008	8	1
SSI-1Δ*yvqE*	0.008	**4**	**0.5**
SSI-1Δ*yvqC*	0.008	**4**	**0.5**
SSI-1Δ*yvqEC*	0.008	**4**	**0.5**
SSI-1Δ*covS*	0.016	8	1
SSI-1Δ*covR*	0.016	**4**	1
SSI-1Δ*covRS*	0.016	**4**	1
SSI-1Δ*yvqEC*Δ*covRS*	0.008	**1**	**0.5**
Wild-type JRS4	0.016	4	1
JRS4Δ*yvqE*	**0.008**	**2**	**0.5**
JRS4Δ*yvqC*	**0.008**	**2**	**0.5**
JRS4Δ*yvqEC*	**0.008**	**2**	**0.5**
JRS4Δ*covS*	0.016	4	1
JRS4Δ*covR*	0.016	4	1
JRS4Δ*covRS*	0.016	4	1
JRS4Δ*yvqEC*Δ*covRS*	**0.008**	**2**	**0.25**

^*a*^ MICs were determined by broth microdilution method in a 96-well plate. Reduction ≥ 2-fold compared with the wild-type strain is indicated in bold. The experiments were independently repeated 3 times.

### Effect of YvqEC system in HA capsule production

**Fig 6:**
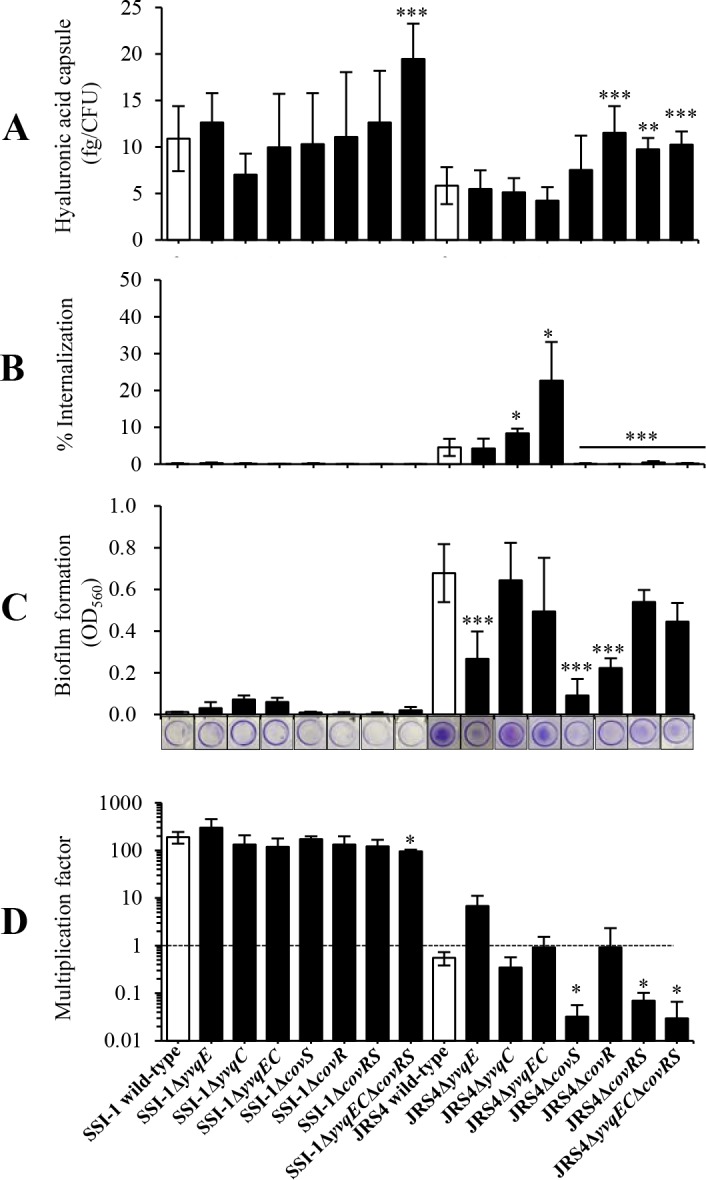
YvqEC and CovRS have a strain-specific role in SSI-1 and JRS4. (A) Hyaluronic acid (HA) production (femtogram; fg/cfu) in SSI-1 and JRS4 wild-type, and their corresponding mutant strains were quantified by Stains-All assay. Data were expressed as the mean and standard deviation from 3 independent experiments. (B) Internalization of SSI-1 and JRS4 wild-type, and their corresponding mutant strains. HeLa cells were infected by SSI-1 and JRS4 wild-type, and their corresponding mutant strains at a multiplicity of infection (MOI) of 100. All data were expressed as the mean and standard deviation from 3 independent experiments in triplicate wells. (C) Biofilm formation of SSI-1 and JRS4 wild-type, and their corresponding mutant strains in C medium under static conditions at 37°C for 24 h. Images show biofilms attaching to the bottom of polystyrene plate after crystal violet staining. Data were expressed as the mean and standard deviation from 3 independent experiments in quadruplicate wells. (D) Lancefield assay of GAS survival in human blood. Multiplication factor is calculated by dividing the number of colony forming units (CFUs) after 3 h of incubation by CFUs in the original inoculum. Horizontal dotted line indicates starting inoculum number. Each strain was tested in 3 individually collected blood samples. Data were expressed as the mean and standard deviation from 3 independent experiments. Asterisks indicate statistically significant differences at *P* < 0.05 (*), *P* < 0.01 (**), and *P* < 0.001 (***) as determined by *t*-test.

The HA capsule is an important virulence factor that enhances the resistance to phagocytosis [32] and is involved in the attachment and invasion of GAS to epithelial cells [33]. In addition, HA capsule production has been shown to be under the control of CovR [34]. Due to HA capsule, GAS colonies have a mucoid morphology when cultivated on agar plates. In this study, wild-type SSI-1 formed a large, mucoid colony, while wild-type JRS4 formed a small, matte colony (S4 Fig). The amount of HA in wild-type SSI-1 was about 2-fold higher than that in wild-type JRS4 (Fig 6A). JRS4Δ*covR* and JRS4Δ*covRS* formed large, mucoid colonies and presented significantly higher amounts (*P* < 0.001 and *P* < 0.01, respectively) of HA than colonies of wild-type JRS4. Although SSI-1Δ*yvqEC* and SSI-1Δ*covRS* showed similar HA-production levels compared with wild-type SSI-1, a significant increase (*P* < 0.001) in HA production was observed in SSI-1Δ*yvqEC*Δ*covRS* compared with wild-type SSI-1. Complementation of *yvqEC* and *covRS* in double mutant restored the HA capsule to the wild-type levels (S3G Fig). This result suggested that CovRS and YvqEC have complementary functions in HA capsule production in the SSI-1 strain.

### YvqEC and CovRS systems affect bacterial internalization to HeLa cells in a strain-specific manner

To characterize the role of YvqEC and CovRS during their interaction with human epithelial cells, we examined the internalization of the wild-type and mutant strains to HeLa cells. The internalization of JRS4Δ*yvqC* and JRS4Δ*yvqEC* was significantly higher (*P* < 0.05) than that of wild-type JRS4 (Fig 6B). In addition, lactate dehydrogenase (LDH)-release assays performed with HeLa cells infected with JRS4Δ*yvqC* and JRS4Δ*yvqEC* showed that both strains induced cytotoxicity at the same level as the wild-type strains (S5 Fig), indicating that HeLa cells remains intact during bacteria internalization. However, JRS4Δ*covS*, JRS4Δ*covR*, JRS4Δ*covRS*, and JRS4Δ*yvqEC*Δ*covRS* exhibited low levels of internalization (< 1%, *P* < 0.001). Complementation of *yvqEC* and *covRS* in the double mutant restored the internalization ability to the wild-type levels (S3H Fig). The internalization efficiency of SSI-1 wild-type and the corresponding mutant strains was less than 1%, with no significant differences between them. Therefore, YvqEC and CovRS play a pivotal role in the internalization of bacterial cells to human epithelial cells in a strain-specific manner.

### YvqEC and CovRS affect biofilm formation in a strain-specific manner

To investigate the involvement of YvqEC and CovRS in biofilm formation, SSI-1 and JRS4 were grown in C medium under static conditions for 24 h. We found that the biofilm formation in SSI-1 wild-type and the corresponding mutant strains was lower than that in JRS4 wild-type and the corresponding mutant strains. Biofilm formation significantly reduced (*P* < 0.001) in JRS4Δ*yvqE*, JRS4Δ*covS*, and JRS4Δ*covR* compared with that in wild-type JRS4 (Fig 6C). However, no significant differences were observed in biofilm formation between SSI-1 wild-type and the corresponding mutant strains.

### CovRS, but not YvqEC, promotes GAS survival and multiplication in human blood

We examined whether YvqEC and CovRS contributed to the virulence of GAS. It is known that GAS is able to evade phagocytes and proliferate in human blood [1, 35]. The number of invasive wild-type SSI-1 increased up to 100-fold over the inoculum after 3 h of exposure to human blood, while the number of non-invasive wild-type JRS4 reduced after the same time period (Fig 6D). The fold-increase in bacterial cell numbers was similar between SSI-1 wild-type and the corresponding mutant strains. However, the proliferation of SSI-1Δ*yvqEC*Δ*covRS* significantly decreased (*P* < 0.05). The proliferation and survival of JRS4Δ*covS*, JRS4Δ*covRS*, and JRS4Δ*yvqEC*Δ*covRS* was significantly impaired (*P* < 0.05) in human blood compared with that of wild-type JRS4. Complementation of *yvqEC* and *covRS* in the double mutant restored the multiplication ability to the wild-type levels (S3I Fig). However, there was no significant difference between Δ*covRS* and Δ*yvqEC*Δ*covRS* in SSI-1 and JRS4, indicating that only CovRS, but not YvqEC, plays a significant role in the survival of GAS in human blood.

## Discussion

Bacterial cell wall synthesis is crucial for bacterial cell growth, division, and structure, and penicillin-binding proteins (PBPs) are involved in bacterial cell wall formation. Inhibition of PBPs leads to irregularities in cell wall structure and eventual cell death. The *ftsL* gene is required for the initiation of cell division in a broad range of bacteria. Therefore, FtsL appears to be essential in *S*. *pneumoniae*, *E*. *coli*, and *B*. *subtilis* [36–38]. Cell viability and division in JRS4Δ*yvqE* and JRS4Δ*yvqC* cells were defective, and downregulation of *pbp1B* and *ftsL* expression was observed (S3 Table), suggesting that the YvqEC system may play a role in regulating cell wall synthesis and division under normal growth conditions. It is unclear how *yvqE* and *yvqC* affect cell viability and division under standard culture conditions, and future studies will assess in more detail, as previous reports [9–18] do not indicate that YvqEC, or its homologs, are important for cell viability and division in GAS or other gram-positive bacteria. VicRK system, which is also known as YycFG or WalRK, is highly conserved and is essential for the viability of GAS [39, 40] and other gram-positive bacteria such as *B*. *subtilis* [41], *S*. *aureus* [42], *S*. *pneumoniae* [43], and *S*. *mutans* [44]. It is possible that YvqEC system in JRS4 serves as a regulatory network, with VicRK being responsible for the control of cell viability.

In this study, JRS4Δ*yvqE* and JRS4Δ*yvqC* showed increased cell death rates (10–20%) and formation of aggregates, as determined by microscopic analysis using fluorescent dyes, despite the viability loss of 1–2 log10 cfu/ml, as determined by plate count assays. This discordance in the number of viable cells could be explained by direct microscopic examination and counting of all cells, both living and dead, in contrast with CFU counting, which only considers viable cells on agar plate. However, the greatest disadvantage of the CFU method is that clumps or aggregates of cells can be produced and are then counted as single colonies, resulting in underestimation of the total number of cells.

The ability of GAS to evolve survival mechanisms in distinct host environments is a key feature of its pathogenicity [1]. GAS uses TCSs to adapt to changing host conditions during infection. CovR/S TCS helps GAS to adapt to several stressful conditions [7]. Ihk/Irr TCS is known to assist in the survival of GAS under oxidative stress conditions produced by human polymorphonuclear neutrophils [45]. CiaRH TCS is also involved in oxidative responses, and the lack of CiaH in GAS serotype M1 results in high sensitivity to H_2_O_2_ [46]. The first study of a GAS *yvqE* mutant pointed out its growth reduction under acid stress [9]. However, several stressors other than acid stress were not examined. Here, we provided the results from various stress conditions, which CovRS and YvqEC is involved in conferring stress tolerance to GAS.

As bacterial resistance to antibiotics becomes more common [47], the need for alternative antibacterial therapies arises [48]. Several features of TCSs make them attractive targets for the development of novel therapeutic applications [49]. Our result showed the deletion of *yvqEC* increased the susceptibility to antibiotics compared with that seen for the wild-type strains. Similar results have been observed in the YvqEC homolog of various bacterial species, with *yvqE/C* mutants being more susceptible to antibiotics that interfere with the lipid-II cycle of cell wall synthesis [11, 12, 14, 18, 20]. Interestingly, our results further showed that the double deletion of *yvqEC* and *covRS* greatly increased the susceptibility to a level higher than that of the wild-type and single mutant. Therefore, this evidence strongly suggested that both YvqEC and CovRS could be potential for therapeutic targets against GAS infection.

It was noted that GAS biofilm formation is strain- and serotype-dependent. For example, serotype M6 belonging to FCT type 1 pili exhibited a greater ability to form biofilms than did other GAS strains [50–52], while serotypes M3 lack the ability to form biofilms due to the absence of the Scl1.3 protein [53]. In this study, we showed significantly decreased biofilm formation in JRS4 Δ*covS* and Δ*covR* compared with that in wild-type JRS4 (serotype M6), but not in SSI-1 (serotype M3). However, no differences in biofilm formation were observed in the JRS4Δ*covRS* and JRS4Δ*yvqEC*Δ*covRS* double mutant. Decreased biofilm formation occurred with JRS4Δ*yvqE* because large aggregates of JRS4Δ*yvqE* cells reduced attachment to the polystyrene surface, whereas the complemented strains had a non-aggregate phenotype that restored biofilm formation (S2D Fig). Thus, JRS4Δ*yvqE* affect not only to the abnormality of cell division but also to biofilm formation. The reason why only JRS4Δ*yvqE* affect to the multiple phenotype of GAS is still unclear.

GAS mucoid strains have been associated with invasive infection. The capsule of GAS is an important virulence factors that confers resistance to phagocytosis [32]. Here, we showed that the highly encapsulated invasive wild-type SSI-1 exhibited a greater ability to survive and multiply in human blood than the unencapsulated wild-type JRS4. Compared with wild-type strains, JRS4Δ*covR*, JRS4Δ*covRS*, and JRS4 Δ*yvqEC*Δ*covRS* that produced higher levels of HA capsule, but their ability to survive in human blood was significantly impaired. These results do not support that the resistance to phagocytosis increases with the amount of HA capsule [32]. We speculate that HA capsule, and perhaps other genes are co-ordinately regulated in GAS survival in blood [35]. In contrast to the M1 GAS study [9], our study did not reveal the Δ*yvqE* to have decreased ability to survive in human blood. Meanwhile, it has been shown that large aggregate cells of GAS are more difficult to uptake by host phagocytes than single cells or single chains [54].

**Fig 7:**
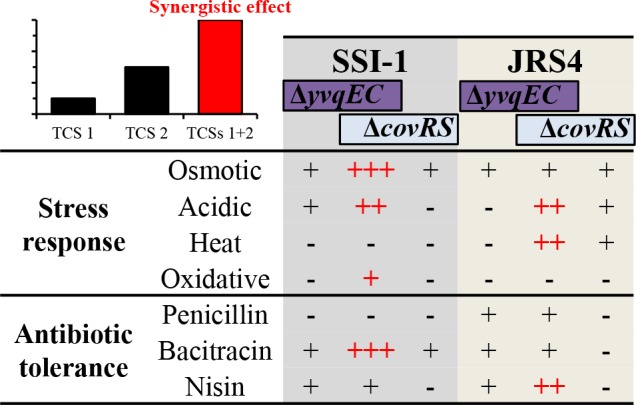
The summary of synergistic effects. The interaction of YvqEC and CovRS TCSs to produce a combined effect is greater than the sum of their individual effects. Red colour (+) indicates synergistic effect of Δ*yvqEC*Δ*covRS*.

This is the first study to report synergistic effect of YvqEC and CovRS systems that the Δ*yvqEC*Δ*covRS* double mutant both SSI-1 and JRS4 strains exceeded the expectations from the additive effects. This double mutant exhibited a greater impact upon stress response and antibiotic susceptibility, providing the hypersensitive phenotype than Δ*yvqEC* and Δ*covRS* mutants (Fig 7). Although further studies with regard to the function, such as transcriptomic or proteomic analysis, are required to elucidate the exact role of Δ*yvqEC*Δ*covRS*. We believe our finding this study provides much useful information for further study toward a comprehensive understanding of the YvqEC and CovRS. It has been reported that *covR* inactivation affects the expression of six TCSs in GAS M1 strain in the stationary growth phase [8]. Thus, there are the interactions between multiple TCSs. However, understanding how TCSs regulatory networks interaction is limited. It would be interesting to determine the precise function of these YvqEC and CovRS interactions and the interactions to other TCSs, remain a key open question.

Overall, this study provides new insights into the impact of poorly characterized YvqEC TCS, provides valuable clues for unveiling the synergistic effect of YvqEC and CovRS TCSs in GAS and their potential role as novel therapeutic targets against GAS infection.

## Supporting Information

S1 Fig Image of standing overnight THY broth culture. Representative images showing bacterial sedimentation of SSI-1Δ*yvqE* and JRS4Δ*yvqE* at the bottom of the tubes in standing overnight THY broth cultures.
(TIF)

S2 Fig Characteristic analysis of the JRS4Δ*yvqE* and JRS4Δ*yvqC* strains. (A) Cell viability of JRS4Δ*yvqE*-complemented and (B) JRS4Δ*yvqC*-complemented strains. All results are shown as the mean and standard deviation from 3 independent experiments. (C) Representative images of LIVE/DEAD-stained JRS4Δ*yvqE-* and JRS4Δ*yvqC*-complemented strains grown in THY at an OD_600_ of 0.8. Magnification, ×60. (D) Biofilm formation of JRS4Δ*yvqE*-complemented strain in C medium under static conditions at 37°C for 24 h. Data were expressed as the mean and standard deviation. Asterisks indicate statistically significant differences compared to the results for the wild-type strain: *P < 0.001 as determined by *t*-test.
(TIF)

S3 Fig Characteristic analysis of the Δ*yvqEC*Δ*covRS*-complemented strain. (A-C) SSI-1Δ*yvqEC*Δ*covRS*-complemented strain grown under osmotic, acidic, and oxidative stress conditions. (D-F) JRS4Δ*yvqEC*Δ*covRS*-complemented strain grown under osmotic, acidic, and heat stress conditions. (G) Hyaluronic acid (HA) production (femtogram; fg/cfu) of SSI-1 and JRS4 Δ*yvqEC*Δ*covRS*-complemented strains. (H) Internalization of JRS4Δ*yvqEC*Δ*covRS*-complemented strain. (I) Multiplication in human blood of SSI-1 and JRS4 Δ*yvqEC*Δ*covRS*-complemented strains. All data were expressed as the mean and standard deviation from 3 independent experiments. Asterisk indicates statistically significant differences at *P* < 0.05 (*) as determined by *t*-test.
(TIF)

S4 Fig Colony morphology of SSI-1 and JRS4 wild-type and the corresponding mutant strains. Colony morphology of SSI-1 and JRS4 wild-type and the corresponding mutant strains grown on THY agar plate cultured overnight. Scale bars, 2 mm.
(TIF)

S5 Fig Cytotoxicity of GAS strains to the HeLa cells. Cytotoxicity on HeLa cells was evaluated by measuring the release of lactate dehydrogenase (LDH) after 4 h of incubation with the bacteria. All data were expressed as the mean and standard deviation from 3 independent experiments.
(TIF)

S1 Table GAS strains and plasmid used in this study. (PDF)

S2 Table Primers used in this study. (PDF)

S3 Table Cell division and cell wall synthesis-related genes that were differentially expressed at OD_600_ of 0.8 in the mutant strains compared to the JRS4 wild-type. All data were expressed as the mean expression ratio from 3 independent experiments. The asterisk (*) indicates down-regulated ≥ 2-fold in the mutant strain with a statistically significant difference between the wild-type and mutant strain (*P* < 0.05) by *t*-test.
(PDF)
