# Supplementary figures and images for: YvqE and CovRS of Group A Streptococcus Play a Pivotal Role in Viability and Phenotypic Adaptations to Multiple Environmental Stresses
Source: PLoS One. 2017 Jan 25;12(1):e0170612. doi: 10.1371/journal.pone.0170612 (PMC5266302; doi:10.1371/journal.pone.0170612)

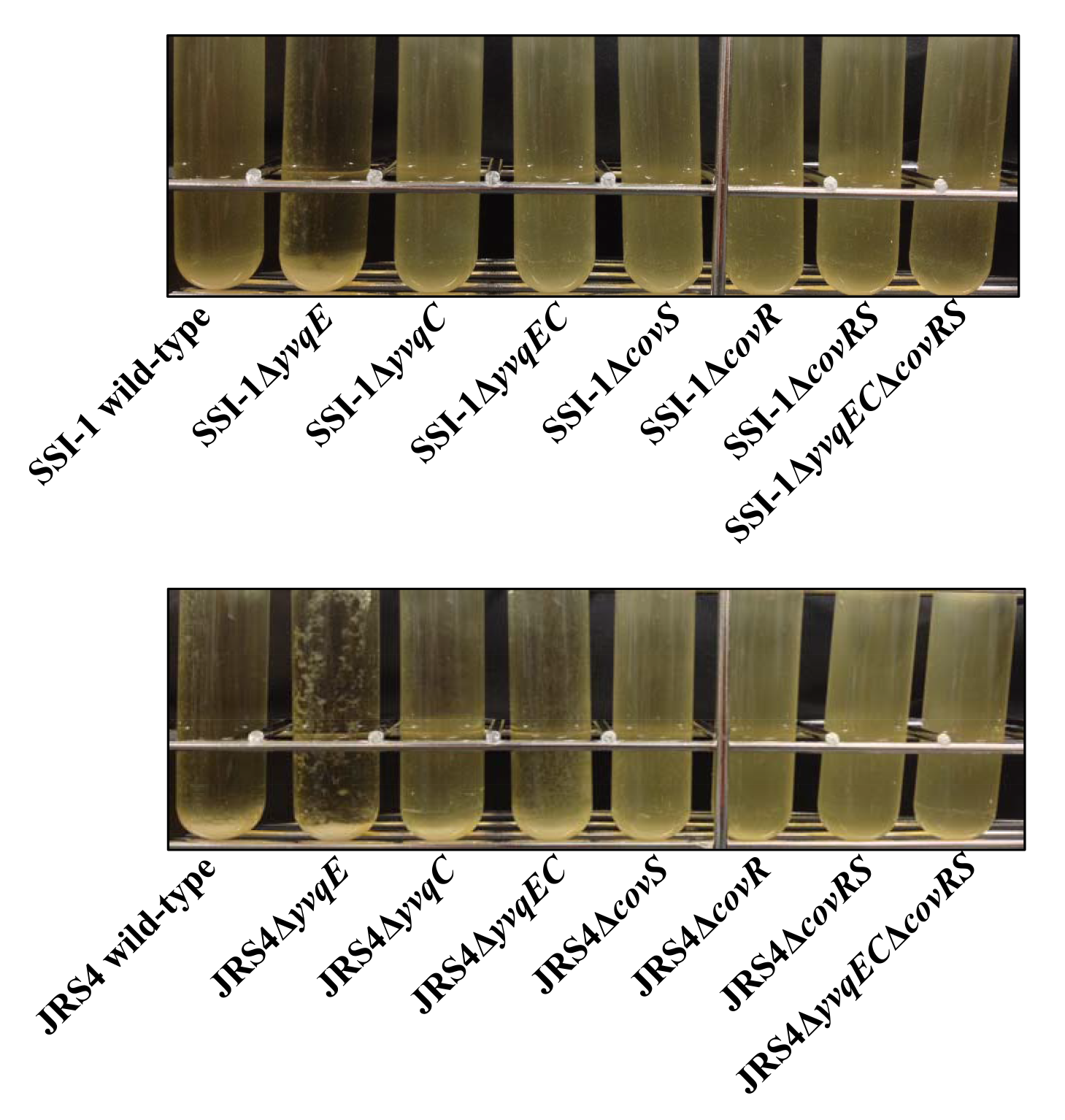

Supplement: S1 Fig — Representative images showing bacterial sedimentation of SSI-1ΔyvqE and JRS4ΔyvqE at the bottom of the tubes in standing overnight THY broth cultures. (TIF) [file pone.0170612.s001.tif]

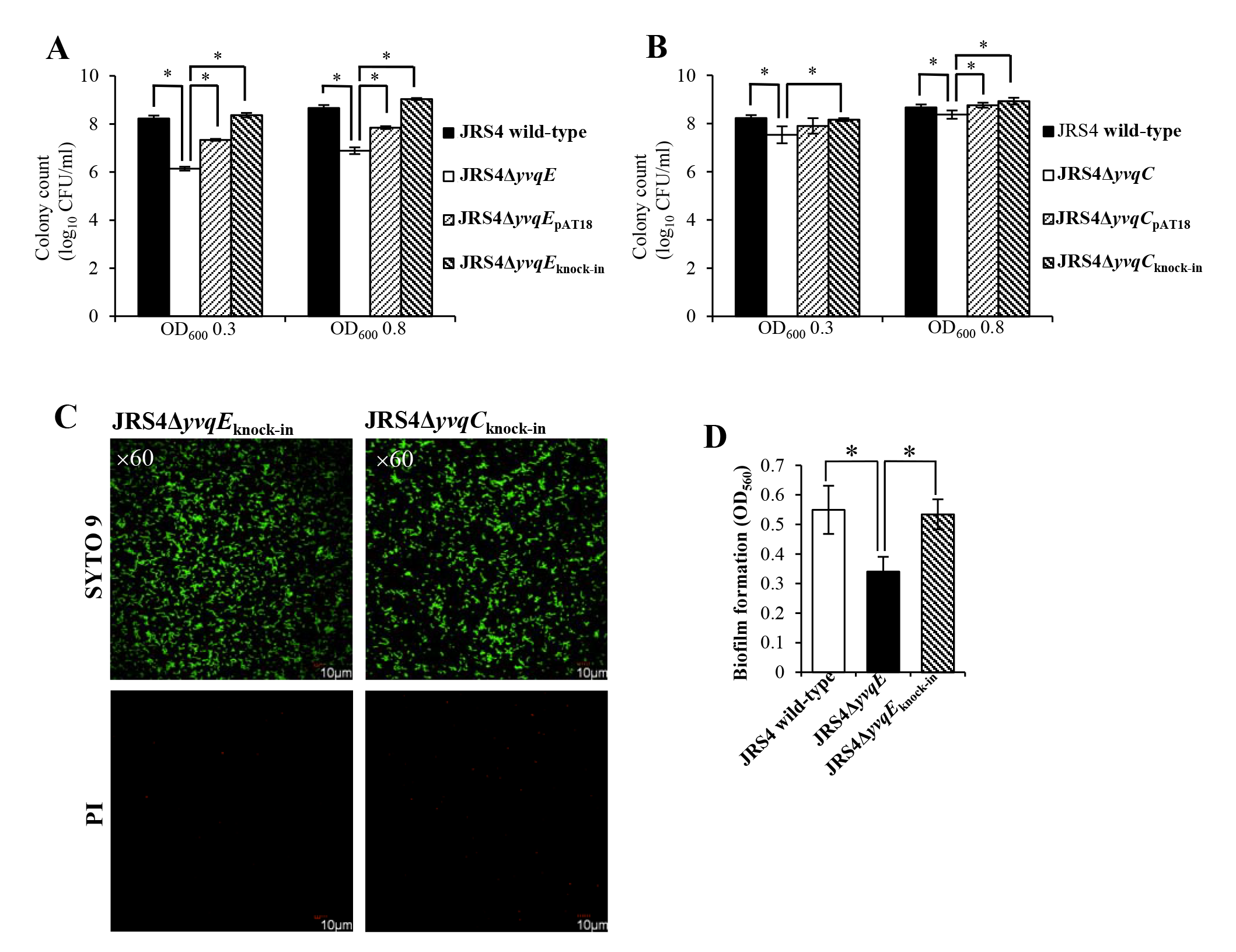

Supplement: S2 Fig — (A) Cell viability of JRS4ΔyvqE-complemented and (B) JRS4ΔyvqC-complemented strains. All results are shown as the mean and standard deviation from 3 independent experiments. (C) Representative images of LIVE/DEAD-stained JRS4ΔyvqE- and JRS4ΔyvqC-complemented strains grown in THY at an OD600 of 0.8. Magnification, ×60. (D) Biofilm formation of JRS4ΔyvqE-complemented strain in C medium under static conditions at 37°C for 24 h. Data were expressed as the mean and standard deviation. Asterisks indicate statistically significant differences compared to the results for the wild-type strain: *P < 0.001 as determined by t-test. (TIF) [file pone.0170612.s002.tif]

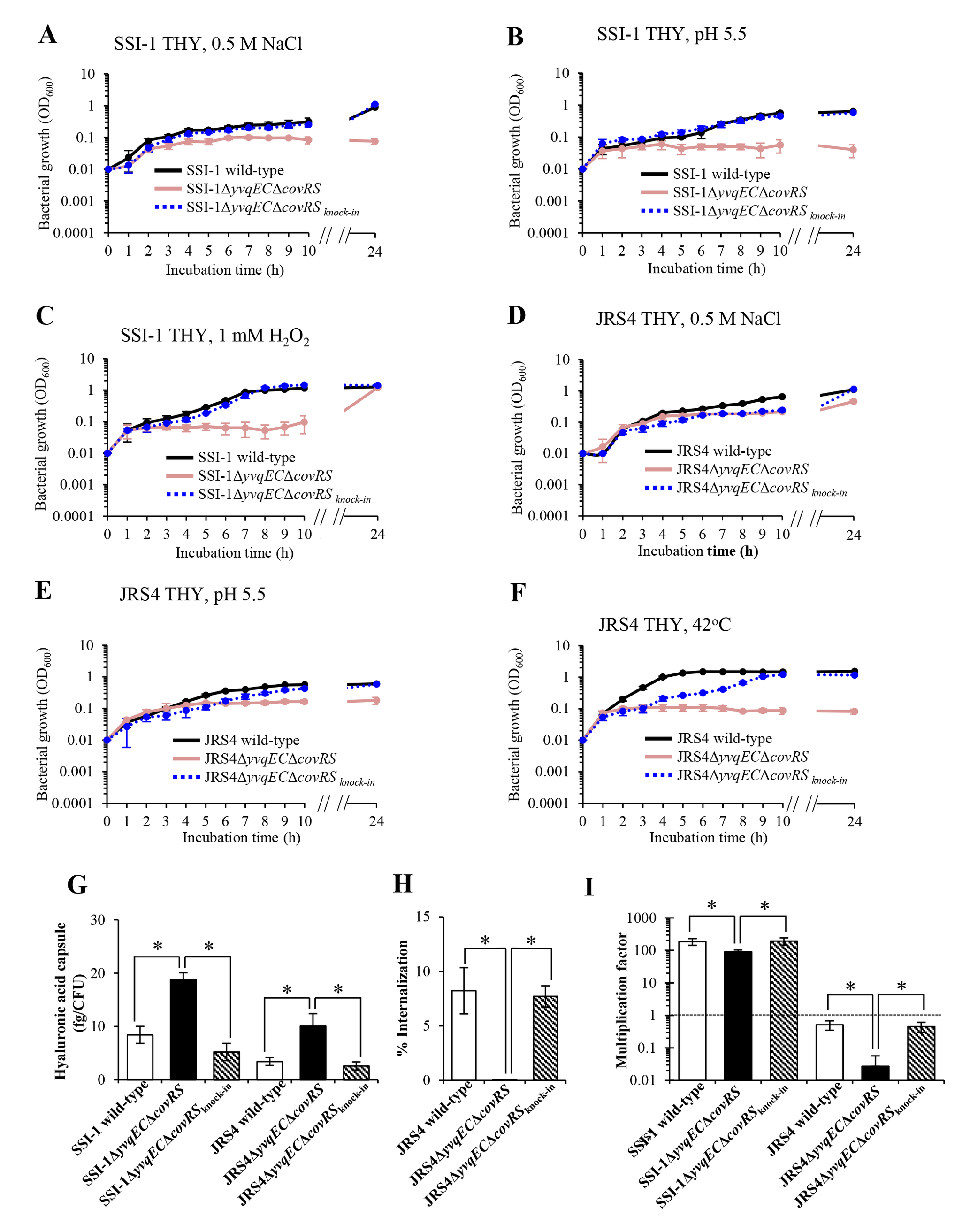

Supplement: S3 Fig — (A-C) SSI-1ΔyvqECΔcovRS-complemented strain grown under osmotic, acidic, and oxidative stress conditions. (D-F) JRS4ΔyvqECΔcovRS-complemented strain grown under osmotic, acidic, and heat stress conditions. (G) Hyaluronic acid (HA) production (femtogram; fg/cfu) of SSI-1 and JRS4 ΔyvqECΔcovRS-complemented strains. (H) Internalization of JRS4ΔyvqECΔcovRS-complemented strain. (I) Multiplication in human blood of SSI-1 and JRS4 ΔyvqECΔcovRS-complemented strains. All data were expressed as the mean and standard deviation from 3 independent experiments. Asterisk indicates statistically significant differences at P < 0.05 (*) as determined by t-test. (TIF) [file pone.0170612.s003.tif]

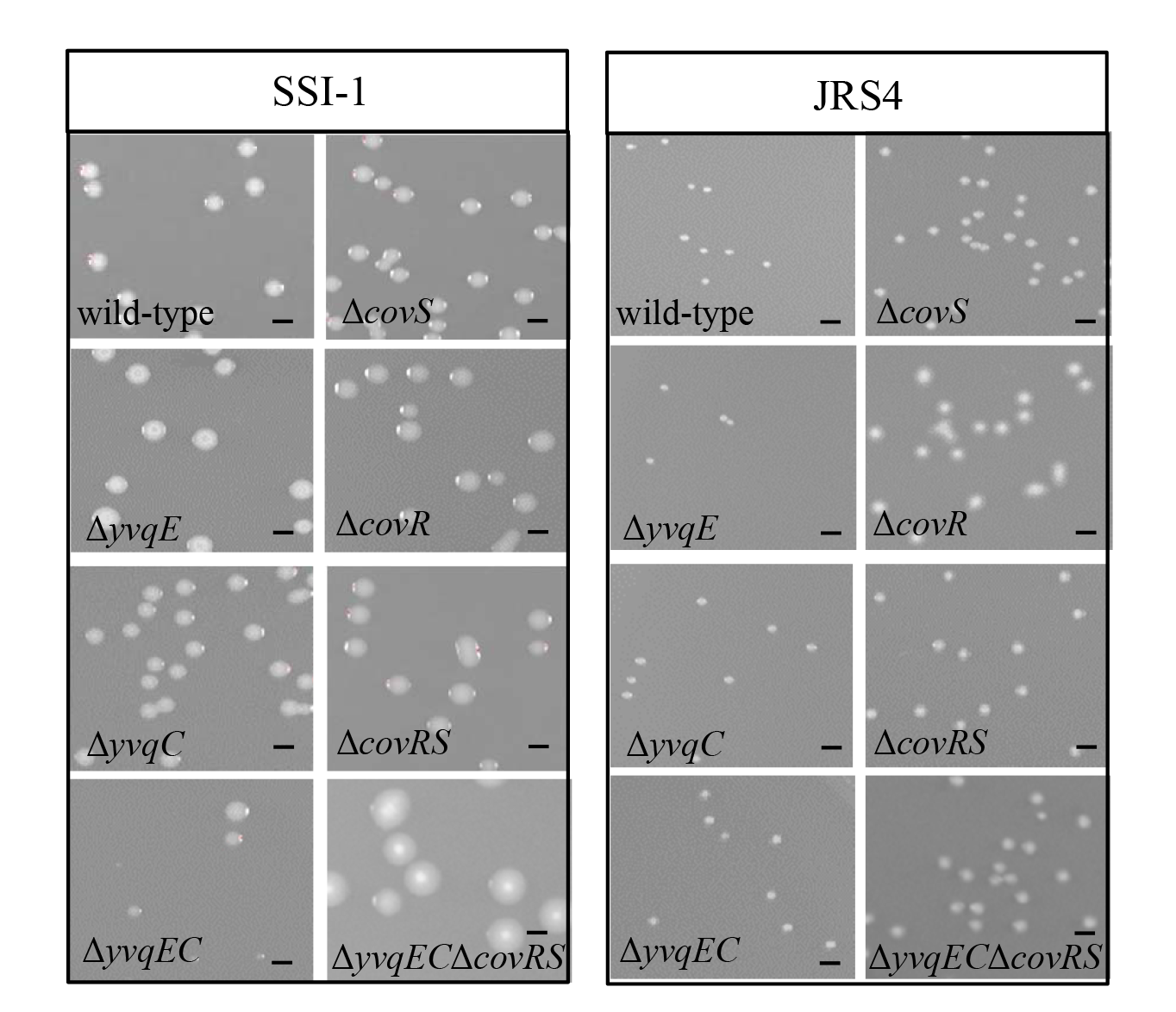

Supplement: S4 Fig — Colony morphology of SSI-1 and JRS4 wild-type and the corresponding mutant strains grown on THY agar plate cultured overnight. Scale bars, 2 mm. (TIF) [file pone.0170612.s004.tif]

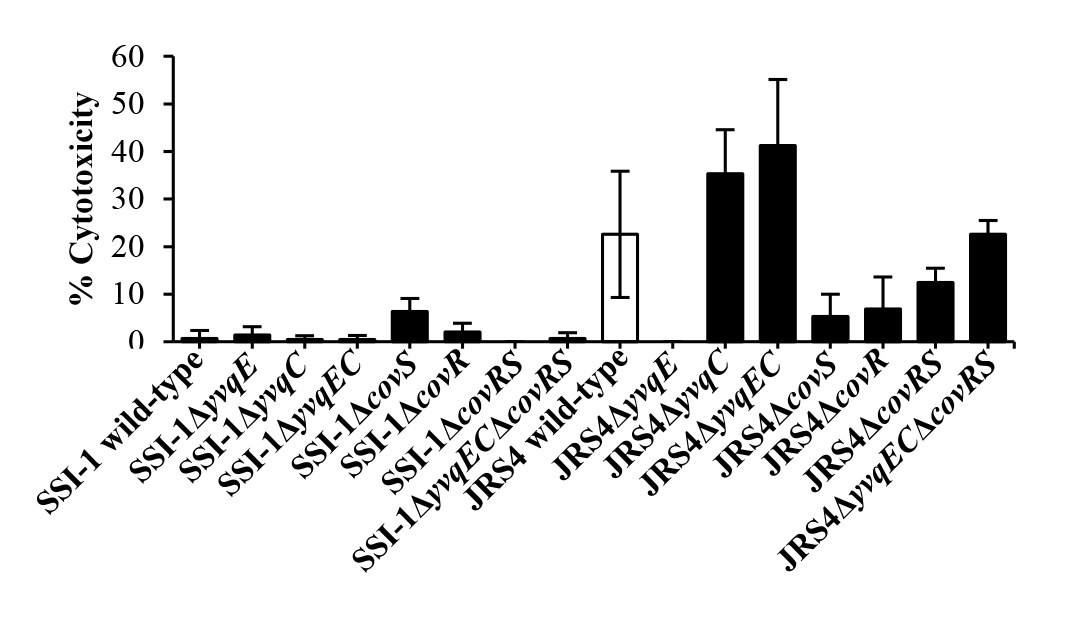

Supplement: S5 Fig — Cytotoxicity on HeLa cells was evaluated by measuring the release of lactate dehydrogenase (LDH) after 4 h of incubation with the bacteria. All data were expressed as the mean and standard deviation from 3 independent experiments. (TIF) [file pone.0170612.s005.tif]
