# Supplementary material for: YvqE and CovRS of Group A Streptococcus Play a Pivotal Role in Viability and Phenotypic Adaptations to Multiple Environmental Stresses
Source: PLoS One. 2017 Jan 25;12(1):e0170612. doi: 10.1371/journal.pone.0170612 (PMC5266302; doi:10.1371/journal.pone.0170612)
Supplement: S1 Table — (PDF) [file pone.0170612.s006.pdf]

Strain or plasmid	Relevant features	Reference/ Source
GAS strains		
SSI-1	Wild-type SSI-1 (serotype M3) was isolated from a patient with streptococcal toxic shock syndrome (STSS) in Japan, 1994.	[22]
SSI-1Δ*yvqE*	*yvqE* (histidine kinase) deletion in SSI-1 background, Spc^S	This study
SSI-1Δ*yvqC*	*yvqC* (response regulator) deletion in SSI-1 background, Spc^S	This study
SSI-1Δ*yvqEC*	*yvqEC* double-deletion in SSI-1 background, Spc^S	This study
SSI-1Δ*covS*	*covS* (histidine kinase) deletion in SSI-1 background, Spc^S	This study
SSI-1Δ*covR*	*covR* (response regulator) deletion in SSI-1 background, Spc^S	This study
SSI-1Δ*covRS*	*covRS* double-deletion in SSI-1 background, Spc^S	This study
SSI-1Δ*yvqEC*Δ*covRS*	*yvqEC* double-deletion in SSI-1Δ*covRS* background, Spc^S	This study
SSI-1Δ*yvqEC*Δ*covRS* $_{\text{knock-in}}$	Knock-in complemented strain of SSI-1Δ*yvqECΔcovRS* with vector pSET4s, Spc^S	This study
JRS4	Wild-type JRS4 (serotype M6) is a spontaneous streptomycin-resistant derivative of serotype M6 strain D471	[23]
JRS4Δ*yvqE*	*yvqE* (histidine kinase) deletion in JRS4 background, Spc^S	This study
JRS4Δ*yvqC*	*yvqC* (response regulator) deletion in JRS4 background, Spc^S	This study
JRS4Δ*yvqEC*	*yvqEC* double-deletion in JRS4 background, Spc^S	This study
JRS4Δ*covS*	*covS* (histidine kinase) deletion in JRS4 background, Spc^S	This study
JRS4Δ*covR*	*covR* (response regulator) deletion in JRS4 background, Spc^S	This study
JRS4Δ*covRS*	*covRS* double-deletion in JRS4 background, Spc^S	This study
JRS4Δ*yvqEC*Δ*covRS*	*yvqEC* double-deletion in JRS4Δ*covRS* background, Spc^S	This study
JRS4Δ*yvqE* $_{\text{pAT18}}$	Complemented strain of JRS4Δ*yvqE* with vector pAT18-recA, Em^r	This study
JRS4Δ*yvqE* $_{\text{knock-in}}$	Knock-in complemented strain of JRS4Δ*yvqE* with vector pSET4s, Spc^S	This study
JRS4Δ*yvqC* $_{\text{pAT18}}$	Complemented strain of JRS4Δ*yvqC* with vector pAT18-recA, Em^r	This study
JRS4Δ*yvqC* $_{\text{knock-in}}$	Knock-in complemented strain of JRS4Δ*yvqC* with vector pSET4s, Spc^S	This study
JRS4Δ*yvqEC*Δ*covRS* $_{\text{knock-in}}$	Knock-in complemented strain of JRS4Δ*yvqECΔcovRS* with vector pSET4s, Spc^S	This study
Plasmids		
pSET4s	*E. coli-S. suis* shuttle vector for gene replacement; Spc^r	[26]
pAT18-recA	Shuttle expression vector under recA promoter; Em^r	[27]

Spc^S, Spectinomycin sensitive; Spc^r, Spectinomycin resistant; Em^r, Erythromycin resistant.
