# Supplementary material for: YvqE and CovRS of Group A Streptococcus Play a Pivotal Role in Viability and Phenotypic Adaptations to Multiple Environmental Stresses
Source: PLoS One. 2017 Jan 25;12(1):e0170612. doi: 10.1371/journal.pone.0170612 (PMC5266302; doi:10.1371/journal.pone.0170612)
Supplement: S2 Table — (PDF) [file pone.0170612.s007.pdf]

Gene	Primer name	Sequence (5' – 3')	Application	Reference/ Source
yvqE	SPs0495upper800fwd	tcgagctcggtacccTAGTCTCATGTTTTACTAGCATGC	Mutant construction, complementation	This study
	SPs0495upper800rev	cacttttatcCGCCTATCCACCTCAACATTTC	Mutant construction	This study
	SPs0495down800fwd	gataggcgGATAAAAGTGATATTGGTCGATG	Mutant construction	This study
	SPs0495down800rev	ctctagaggatccccCCTTCAAAGCCTTAAATACTC	Mutant construction, complementation	This study
yvqC	SPs0496upper800fwd	tcgagctcggtacccGATTATTATGGATGATAACAGTAAGCG	Mutant construction, complementation	This study
	SPs0496upper800rev	tattatagcaCTCCCTTCACTATCGGCAG	Mutant construction	This study
	SPs0496down800fwd	gaagggagTGCTATAATATAGGGTATAGCAAATTG	Mutant construction	This study
	SPs0496down800rev	ctctagaggatccccTCTTCTTCAACTTTTTTTGTGATAAAATC	Mutant construction, complementation	This study
yvqEC	SPs0495/0496upper800fwd	tcgagctcggtacccTAGTCTCATGTTTTACTAGCATGC	Mutant construction, complementation	This study
	SPs0495/0496upper800rev	tattatagcaCGCCTATCCACCTCAACATTTC	Mutant construction	This study
	SPs0495/0496down800fwd	gataggcgTGCTATAATATAGGGTATAGCAAATTG	Mutant construction	This study
	SPs0495/0496down800rev	ctctagaggatccccTCTTCTTCAACTTTTTTTGTGATAAAATC	Mutant construction, complementation	This study
covS	SPs1614upper800fwd	tcgagctcggtacccAGAAGACTATTTGACCATTAGAAC	Mutant construction	This study
	SPs1614upper800rev	taagctactATGACTTATTTCTCACGAATAAC	Mutant construction	This study
	SPs1614down800fwd	aataagtcatAGTAGCTTAAGGCATTGTTAATGTG	Mutant construction	This study
	SPs1614down800rev	ctctagaggatccccGGCTGCGTCACTTCCAAT	Mutant construction	This study
covR	SPs1615upper800fwd	tcgagctcggtacccTGTTAGCTATTTCCGAAATCAG	Mutant construction	This study
	SPs1615upper800rev	ccatatgacTTATACCAACCCTTATCCTC	Mutant construction	This study
	SPs1615down800fwd	gttggtataaGTCATATGGAAAATCAGAAACAAAAAC	Mutant construction	This study
	SPs1615down800rev	ctctagaggatccccCTCCAATTTGTCTAACATATTGTC	Mutant construction	This study
covRS	SPs1614/1615upper800fwd	tcgagctcggtacccTGTTAGCTATTTCCGAAATCAG	Mutant construction, complementation	This study
	SPs1614/1615upper800rev	taagctactTTATACCAACCCTTATCCTC	Mutant construction	This study
	SPs1614/1615down800fwd	gttggtataaAGTAGCTTAAGGCATTGTTAATGTG	Mutant construction	This study
	SPs1614/1615down800rev	ctctagaggatccccGGCTGCGTCACTTCCAAT	Mutant construction, complementation	This study
yvqE	yvqE-1352_fwd	cggtaagggcgcagcATGAAAAAACGTTACTATGCTCTTGTTTGG	Complementation	This study
	yvqE-1352_rev	ctatgaccatgattacgTTACTCATCGTCATCTCCCTTCAC	Complementation	This study
yvqC	yvqC-1351_fwd	cggtaagggcgcagcATGAGTAAGATAAAAGTGATATTGGTCGATG	Complementation	This study
	yvqC-1351_rev	ctatgaccatgattacgTTAATTATCGTCTTGGGGGACTAAATG	Complementation	This study
proS	proS_fwd	GGGTGGTTCTTGACAAGTCTATTGCG	qRT-PCR	[28]
	proS_rev	TTCTGCCAAGGCATCTTCAGCA		
pbp1A	pbp1A_fwd	AACGTGCCATGAAAGAAACC	qRT-PCR	[28]
	pbp1A_rev	AAGTTTTCGTCTGGCGCTAA		
pbp2A	pbp2A_fwd	ACGCCACAACACAATCAAAA	qRT-PCR	[28]
	pbp2A_rev	AATTGGACGTTGAGGAGACG		
pbp1B	pbp1B_fwd	AGATTTAACGGTGCCACAGG	qRT-PCR	[28]
	pbp1B_rev	ACTATTGCACTTCCCGGTTG		
ftsL	ftsL_fwd	CAGCGATTACGATGGCAGTA	qRT-PCR	[28]
	ftsL_rev	AAGCCTGCTTTACCAGCAAT		

Nucleotides shown in lowercase letters denote extra sequences for restriction endonuclease recognition sites
