# Supplementary material for: YvqE and CovRS of Group A Streptococcus Play a Pivotal Role in Viability and Phenotypic Adaptations to Multiple Environmental Stresses
Source: PLoS One. 2017 Jan 25;12(1):e0170612. doi: 10.1371/journal.pone.0170612 (PMC5266302; doi:10.1371/journal.pone.0170612)
Supplement: S3 Table — All data were expressed as the mean expression ratio from 3 independent experiments. The asterisk (*) indicates down-regulated ≥ 2-fold in the mutant strain with a statistically significant difference between the wild-type and mutant strain (P < 0.05) by t-test. (PDF) [file pone.0170612.s008.pdf]

Gene name	function	log2 fold-change	
		Δ*yvqE*/wild-type	Δ*yvqC*/wild-type
penicillin binding protein; *pbp1A*	Cell wall synthesis	0.516	0.937
penicillin binding protein; *pbp2A*	Cell wall synthesis	-0.169	-0.174
penicillin binding protein; *pbp1B*	Cell wall synthesis	-1.513*	-1.070*
ftsL	Cell division	-1.307*	-0.318
